# Quantitative Proteomics Analysis of the Hepatitis C Virus Replicon High-Permissive and Low-Permissive Cell Lines

**DOI:** 10.1371/journal.pone.0142082

**Published:** 2015-11-06

**Authors:** Fei Ye, Zhongshuai Xin, Wei Han, Jingjing Fan, Bin Yin, Shuzhen Wu, Wei Yang, Jiangang Yuan, Boqin Qiang, Wei Sun, Xiaozhong Peng

**Affiliations:** 1 The State Key Laboratory of Medical Molecular Biology, Department of Molecular Biology and Biochemistry, Institute of Basic Medical Sciences, Chinese Academy of Medical Sciences and Peking Union Medical College, Beijing, China; 2 Division of Hormone, National Institute for Food and Drug Control, Beijing, China; 3 Core facility of instrument, Institute of Basic Medical Sciences, Chinese Academy of Medical Sciences and Peking Union Medical College, Beijing, China; 4 MOH Key Laboratory of Systems Biology of Pathogens, Institute of Pathogen Biology, Chinese Academy of Medical Sciences and Peking Union Medical College, Beijing, China; National Institute of Infectious Diseases, JAPAN

## Abstract

Chronic hepatitis C virus (HCV) infection is one of the leading causes of severe hepatitis. The molecular mechanisms underlying HCV replication and pathogenesis remain unclear. The development of the subgenome replicon model system significantly enhanced study of HCV. However, the permissiveness of the HCV subgenome replicon greatly differs among different hepatoma cell lines. Proteomic analysis of different permissive cell lines might provide new clues in understanding HCV replication. In this study, to detect potential candidates that might account for the differences in HCV replication. Label-free and iTRAQ labeling were used to analyze the differentially expressed protein profiles between Huh7.5.1 wt and HepG2 cells. A total of 4919 proteins were quantified in which 114 proteins were commonly identified as differentially expressed by both quantitative methods. A total of 37 differential proteins were validated by qRT-PCR. The differential expression of Glutathione S-transferase P (GSTP1), Ubiquitin carboxyl-terminal hydrolase isozyme L1 (UCHL1), carboxylesterase 1 (CES1), vimentin, Proteasome activator complex subunit1 (PSME1), and Cathepsin B (CTSB) were verified by western blot. And over-expression of CTSB or knock-down of vimentin induced significant changes to HCV RNA levels. Additionally, we demonstrated that CTSB was able to inhibit HCV replication and viral protein translation. These results highlight the potential role of CTSB and vimentin in virus replication.

## Introduction

Hepatitis C virus (HCV) is a positive-stranded RNA virus that causes acute and chronic hepatitis. A striking feature of HCV infection is the high risk of contracting liver diseases in persistently infected patients, up to 60–80% of infected adults progress to liver cirrhosis and hepatocellular carcinoma [1]. With over 180 million people currently infected, HCV represents a growing world health problem [2]. Although many issues have been addressed since HCV was first identified, the lack of a virus culture system was a serious handicap in the fight against HCV infection. The development of an HCV replicon system enabling HCV subgenomic RNA replication in Huh7 human hepatoma cells allowed the study of mechanisms underlying HCV replication [3]. The initial functional replicon that was previously reported is HCV genotype 1, and efficient replication of this replicon has been accomplished only in limited human hepatocyte-derived cell lines [4–6]. Kato et al. developed an HCV genotype 2a replicon (JFH-1) that replicates efficiently in Huh7 cells and other human hepatocyte-derived cells lines (HepG2 and IMY-N9) [7–9] and nonhepatic cells lines (HeLa and HEK293) [10, 11] without adaptive mutations. Although these cell lines can uptake the HCV subgenomic replicon, the efficiency of replication in cells differs significantly because of host cell permissiveness. In 2005, an efficient virus production system using the JFH1 strain was developed using Huh7-derived cell lines [12, 13]. In this system, Huh7 is the only cell line that enables persistent HCV production without additional host factors [14], although a new human hepatoma cell line (Li23), was recently reported to enable genome-length HCV RNA replication [15, 16]. Other hepatocyte-derived cells, such as HepG2 cells, support the HCV 2a subgenomic replicon with lower efficiency compared to Huh7. HepG2 cells differ by up to two orders of magnitude in their level of permissiveness [9]. To date, there is still no evidence to support robust replication of the HCV genotype 1 subgenomic replicon or 2a genomic replicon in HepG2 cells without the addition of external factors.

The permissiveness of the host cell critically contributes to the different efficiency of RNA replication [17, 18]. However, the mechanisms behind the different levels of permissiveness in the two cell lines are unknown. Evidence suggests that the level of permissiveness is determined by the availability of host cell factor(s) required for RNA replication, presumably limiting replication in cells with low permissiveness [18]. One important finding is that liver-specific microRNA 122 (miR-122) is highly expressed in Huh7 cells and absent in HepG2 cells [19]. MiR-122 can facilitate replication of HCV viral RNA, suggesting one possible cause of the different levels of permissiveness between the two cell lines. Hepatic cell lines transfected with miR-122 were able to support the entire HCV life cycle. However, long-term multi-cycle HCV spread was less efficient in HepG2 cells expressing miR122 compared with Huh7.5.1 cells [16, 20]. In addition to microRNA, a number of other host cell factors may also be involved in facilitating HCV replication or translation.

Proteomic analysis provides a large-scale view of proteins expression in cells or tissues. Therefore, differential proteomic analysis might identify disease-related proteins and provide possible clues to their functions. Proteomic technology has been employed to find potential proteins that associate or interact with HCV [21–24]. Jacobs et al. analyzed HCV replicon-positive and negative Huh7.5.1 cells and identified changes in expression of proteins involved in lipid metabolism [21]. Xun et al. examined the differential expression of proteins in Huh7 cells and Huh7 cells harboring *in vitro*-transcribed full-length HCV 1b RNA (Huh7-HCV) and found 13 differential proteins related to HCV replication [22]. The above reports added new information to the understanding of HCV and may provide new clues for the treatment of HCV infection.

In this study, we investigated key proteins contributing to the permissiveness of different hepatoma cells using proteomic analysis of two cells with different permissiveness for HCV RNA replication. Huh7.5.1 wt cells were ‘cured’ cells derived from R1b cells by treatment with interferon [17], which support higher levels of HCV RNA replication compared to parental Huh7 cells, and HepG2 have a low level of HCV subgenome permission. Identification of differential proteins functions may reveal factors contribute to HCV replication. Using label-free and iTRAQ labeling quantitative proteomic approaches, a total of 680 differentially expressed proteins were identified. QRT-PCR and western blot were used to verify expression levels of these proteins. However, CTSB and vimentin were identified as regulators of HCV replication by protein functional studies, and may therefore be potential molecular targets for HCV treatment.

## Materials and Methods

### Cells and Virus

Huh7.5.1 human hepatoma cells were kindly provided by Francis Chisari (Scripps Research Institute, La Jolla, CA, USA) and were maintained in complete DMEM. HepG2 (ATCC), Hep3b and SMMC7721 were maintained in MEM medium while L-02 were maintained in RPMI1640 medium supplemented with 2 mM L-glutamine, 100 U/ml of penicillin, 100 mg/ml of streptomycin and 10% FBS [25, 26]. Hep3b, SMMC7721 and L-02 were obtained from Cell culture center of Chinese Academy of Medical Sciences and Peking Union Medical College. R1b cells expressing an HCV pooled subgenome replicon were developed in our laboratory as described previously [3, 27] and maintained with an additional 500 μg/mL G418 supplement. To obtain cured Huh7.5.1 wt cells, R1b cells were treated with 1000 IU/ml of IFN-α in the absence of G418. The treatment was continued for 2 weeks with the addition of IFN-α at 3-day intervals. Clearance of HCV RNA in these cured cells was confirmed by qRT-PCR.

### Apparatus

An LTQ Velos Orbitrap and a TripleTOF 5600 mass spectrometer were from Thermo fisher (Bremen, Germany) and ABsciex (Framingham, USA). An ACQUITY UPLC system was purchased from Waters (Milford, MA). An ADVANCE CaptiveSpray source for Thermo and C18 reverse phase (RP) capillary were purchased from Michrom Bioresources (Auburn, CA).

### Reagents

Deionized water from a MilliQ RG ultrapure water system (Millipore, MA) was used at all times. HPLC grade I and formic acid, ammonium bicarbonate, iodoacetamide, DTT, sequencing grade modified trypsin, and protease inhibitor PMSF were purchased from Sigma (St. Louis MO, USA).

### Protein Sample

Cell population were harvested in 50 ml tubes by centrifugation for 5 min at 400 ×g, washed five times with phosphate-buffered saline, and lysed with lysis buffer (7 M urea, 2 M thiourea, 50 mM tris, 50 mM DTE, 1 mM PMSF, 1 mM Rnase and 1 mM Dnase) in a homogenizer on ice. The lysate was centrifuged for 10 min at 12,000×*g*, and the supernatant was collected. The radford method was used to determine the protein concentration.

### Protein Digestion

The protein samples were digested using the filter-aided sample preparation method [28]. Briefly, the protein sample were first reduced with 20 mM DTT at 95°C for 3–5 min, and then washed once with 8M urea on 10Kda filter (Pall) at 14,000 g for 40 min. The mixture was alkylated with 55 mM iodoacetamide for 30 min in the dark, and washed twice with 8 M urea. The protein sample were washed with 50 mM ammonium bicarbonate once, and then digested with trypsin (1 μg/50 μg protein) overnight at 37°C. The peptide mixtures were desalted on an OASIS C18 solid phase extraction column (Waters). All samples were lyophilized for MS analysis. The concentration of peptides was determined by BCA kits at 562 nm.

### iTRAQ labeling

The two digested samples were mixed at equal amount as an internal standard. The two samples and the internal standard were labeled with 115, 116 and 117 iTRAQ. Labeling was performed according to the manufacturer’s protocol (Absciex). The three samples were pooled into one sample at equal peptide amounts and lyophilized.

### LC-MS/MS analysis

#### Label-free approach

The two digested samples were analyzed using an RP C18 capillary LC column from Michrom Bioresources (100 μm×150 mm, 3 μm). The gradient was eluted in 5–30% buffer B1 (0.1% formic acid, 99.9% I; flow rate, 0.5 μL/min) for 100 min. Each sample was run three times. Orbitrap-LTQ-CID mode was used to acquire raw data. Precursors were scanned with an Obitrap analyzer and then fragmented and scanned with an LTQ analyzer. MS data were acquired using the following parameters: 20 data-dependent CID MS/MS scans per every full scan, full scans were acquired in Orbitrap at resolution 60,000, 35% normalized collision energy in CID included internal mass calibration (445.120025 ion as lock mass with a target lock mass abundance of 0%), charge state screening (excluding precursors with unknown charge state or +1 charge state) and dynamic exclusion (exclusion size list 500, exclusion duration 30 s).

#### iTRAQ approach

The pooled mixture from three labeled samples was first fraction with a high-pH RPLC column from Waters (4.6 mm×250 mm, C18, 3 μm). The samples were loaded onto the column in buffer A2 (100% H_2_O, pH = 10). The gradient was eluted in 5–30% buffer B2 (90%CAN; pH = 10, flow rate 1 mL/min) for 60 min. The eluted peptides were collected as one fraction per minute, and the 60 fractions were pooled into 20 samples. Each sample was analyzed using a RP C18 capillary LC column from Michrom Bioresources (100 μm×150 mm, 3 μm). The gradient was eluted in 5–30% buffer B1 (0.1% formic acid, 99.9% I; flow rate, 0.5 μL/min) for 100 min. TripleTOF 5600 was used to analyze the samples. MS data were acquired with the high sensitivity mode using the following parameters: 30 data-dependent MS/MS scans per every full scan; full scans acquired at resolution 40,000 and MS/MS scans at 20,000; 35% normalized collision energy, charge state screening (including precursors with +2 to +4 charge state) and dynamic exclusion (exclusion duration 15 s); MS/MS scan range was 100–1800 m/z and scan time was 100 ms.

### Database search

The MS/MS spectra were searched against the SwissProt human database from the Uniprot website (www.uniprot.org) using Mascot software version 2.3.02 (Matrix Science, UK). Trypsin was chosen as cleavage specificity with a maximum number of allowed missed cleavages of two. Carbamidomethylation I and the iTRAQ 4-plex label were set as fixed modifications for the iTRAQ labeling approach. The searches were performed using a peptide tolerance of 5 ppm and a product ion tolerance of 0.5 Da in a label-free approach, and peptide and product ion tolerance of 0.05 Da in the iTRAQ method. Scaffold was used to further filter the database search results using the decoy database method. A 1% false positive rate at the protein level was used as a filter, and each protein contained at least 2 unique peptides.

### Quantitation analysis

Label-free approach: The raw files from label-free analysis were imported into Progenesis LC-MS (v2.6, Nonlinear Dynamics, UK). A reference run was selected from the wild type sample injections and other runs were aligned to the reference run. The abundance of features among different LC/MS/MS runs was normalized. Alignment was achieved by use of the automated alignment algorithm within Progenesis LC-MS. Features were filtered for charge state (+2 to +5). To integrate MS features with protein identities, MS/MS spectra from all runs were exported and searched against the SwissProt human database using Mascot. The results were imported back into Progenesis LC-MS and the peptide identifications were mapped onto the quantitative peptide data. Peptide quantitative results were converted into protein quantitative results by peptide reassignment with Progenesis LC-MS. Proteins with a fold change > 2 and p-value < 0.05 were considered as significant.

The database search results from iTRAQ-labeling approach were imported into Scaffold for further analysis. After filtering the results by above database search filter, the peptide abundances in different reporter ion channels of MS/MS scan were normalized. The protein abundance ratio was based on unique peptide results. Proteins with a fold change > 2 were considered significant.

### GO functional analysis

Differential proteins (Table 1) identified by two proteomic analysis approaches were assigned their gene symbol via the Panther database (http://www.pantherdb.org/). Protein classification was performed based on functional annotations using Gene Ontology (GO) for biological process and molecular function. When more than one assignment was available, all of the functional annotations were considered in the results.

**Table 1:** The qualitative and quantitative results from the label-free and 4-plex iTRAQ methods.

Method	Qualitative result			Quantitative result		
	Protein	peptide	Spectrum	total	differential	common
Label-free	1467	8118	52993	1416	393	114
iTRAQ	4944	38,191	215,287	4900	457	114

### IPA network analysis

The SwissProt accession numbers of differential proteins (Table 1) were inserted into the Ingenuity Pathway Analysis (IPA) software (Ingenuity Systems, Mountain View, CA). This software categorizes gene products based on the location of the protein within cellular components and suggest possible biochemical, biological and molecular functions. Proteins were mapped to genetic networks available in the Ingenuity and other databases and ranked by score. These genetic networks describe functional relationships between gene products based on known interactions in literature. The newly formed networks were associated with known biological pathways with the IPA software.

### Real-time qRT-PCR analysis

Differential proteins were verified by qRT-PCR analysis. Briefly, cells were cultured for RNA isolation using Trizol reagent (Invitrogen, USA) following the manufacturer’s protocol. Then 1 μg total RNA was reverse transcribed to cDNA using random primers and first-strand cDNA synthesis superMix (TransGen biotech, China). QRT-PCR was performed on a C1000 Thermal cycler (Bio-rad, USA) with SYBR Green Mix (Takara Bio INC, Japan) as a previously described method [27]. The qRT-PCR procedure was as follow: an initial step of 1 min at 95°C, followed 40 cycles for 10 s at 95°C and 30 s at 60°C. The disassociation analysis was routinely carried out by acquiring a fluorescent reading from 55°C to 95°C. All qRT-PCR products were verified by melting curve analysis. Primers sequences are provided in the supporting information. The fold-relative enrichment was quantified with normalization to the GAPDH level. Each experiment was repeated at least three times.

### Over-expression or knockdown of differential proteins

To test the key differential proteins function to HCV subgenome replication, R1b cells were transfected with 20 nM siRNAs targeting GSTP1 [29], UCHL1 [30], vimentin [31] or plasmids over-expressing CES1, PSME1, CTSB using Lipofection 2000 (Invitrogen, USA) according to the manufacturer’s protocol. The primers of over-expression for CTSB, CES1, vimentin, and PSME1 were described in Table 2. Western blot and qRT-PCR analysis were used to analyze levels of each proteins, β-actin, HCV RNA, and GADPH mRNA.

**Table 2:** Primers used in this study.

Description	Primer	sequence(5'-3')
pcDNA4.0-PSME1	Fwd	CCGCTCGAGATGGCCATGCTCAGGGTCCAGC
	Rev	GCTCTAGATCATTCCCTTTG
pcDNA4.0-CTSB	Fwd	CCGCTCGAGATGTGGCAGCTCTGGGCCTC
	Rev	GCTCTAGATTAGATCTTTTCCCAGTACTGATCGG
pcDNA4.0-CES1	Fwd	CGGAATTCATGTGGCTCCGTGCCTTTATC
	Rev	CCGCTCGAGTCACAGCTCTATGTGTTCTG
pcDNA4.0-vimentin	Fwd	GGAATTCATGTCCACCAGGTCCGTGTC
	Rev	GCTCTAGATTATTCAAGGTCATCGTGATG

### Cell Culture-derived HCV (HCVcc) and Luciferase reporter assays

To demonstrate the key differential proteins to HCVcc replication, Huh7.5.1 cells were transfected with 20 nM siRNA, and a second siRNA transfection was processed after 24 h. Then cells were infected with Jc1-Luc HCVcc after 48 h, and Luciferase activity was measured 48 h later (Promega, USA). The production and infection procedures of HCV JFH1 were described previously [32]. And the production of Jc1-luc chimeric HCV cell culture (HCVcc) was performed as previously described [33].

### *In vitro* transcription

To verified the key differential proteins to HCV subgenome replication, HepG2 cells were transfected with 20 nM siRNAs targeted to CTSB [34] or over-expression plasmids of vimentin, and cells were transfected with HCV 1b RNA using Interferin (Polyplus-transfection Inc, USA) 12 h later and harvested for protein extraction and total mRNA isolation after 24 and 48 h. Non-related control siNC (Genepharma, China) and pcDNA3.1 were used as negative controls.

ScaI-digested replicon constructs were further purified with ethanol and sodium acetate and used as templates for *in vitro* RNA synthesis using the RiboMAX^™^ Large Scale RNA Production System-T7. Synthesized HCV subgenomic RNA was treated with Dnase I (Promega, USA) followed by acid phenol extraction to remove any remaining template DNA.

### Western blot analysis

Western blot was performed following a previously described method [35]. The dilution of primary antibodies as below: UCHL1 (diluted in 1:1000, Enzo life sciences), GSTP1 (diluted in 1:1000, Calbiochem), Vimentin and CES1 (diluted in 1:500, Santa Cruz Biotechnology), CTSB and PSME1 (diluted in 1:500, Epitomics Biotechnology). The HCV core (diluted in 1:1000, Thermo Fisher Scientific), Mouse monoclonal β-actin antibody (diluted in 1:3000, Sigma). HRP-linked anti-IgG ECL reagent (Amersham Biosciences, UK) was used as the substrate for detection, and the membrane was exposed to an X-ray film for visualization.

### Statistical Analysis

The qRT-PCR and Western blot assay were performed in triplicate. The data were analyzed using a two-tailed paired Student’s t test, and Values with *P<0*.*05* were considered to be significant.

## Results

### Proteomic analysis to detect differentially proteins

To achieve a comprehensive differential proteomic analysis, two quantitative approaches were used in this work. The first is a label-free method based on peptide peak area, whereas the second is a labeled method based on iTRAQ labeling.

#### Label-free approach

The two samples (Huh7.5.1 wt and HepG2 cells) were analyzed by 1D LC-MS/MS. A total of 6 LC-MS/MS runs (3 for each cell line) were imported into Progenesis LC-MS and subjected to alignment with a reference run. MS feature detection was filtered by peptide charge state (+2 to +5) and resulted in 40,444 features. The features from the MS/MS scan were exported from Progenesis LC-MS for a Mascot database search. A total of 103,610 MS/MS spectra were searched against the SwissProt database and 52,993 spectra were identified. The results were further filtered by Scaffold with 1% FDR at the protein level and 2 unique peptides for each identification, resulting in 8118 peptides and 1467 proteins (Table 1, detailed data in S1 Table). A total of 1416 proteins could be used for quantitative analysis. The mean protein abundance CV was 11.57%.

S1 Fig compared the protein abundance ratio of any two inter-run analyses using linear regression. The abundance ratio of replicate experiments performed very well with R^2^ above 0.99, which showed the good experimental reproducibility of this study.

To evaluate the false positive frequency in protein abundance changes based on peptide peak area criteria, the three runs for each sample were mutually compared. The average false positive frequency of inter-run analysis was 1.71% (false positive frequency is the protein number with an abundance change greater than 2-fold divided by the total protein number in any two runs of a given sample; detailed data for each sample not shown).

**Fig 1:**
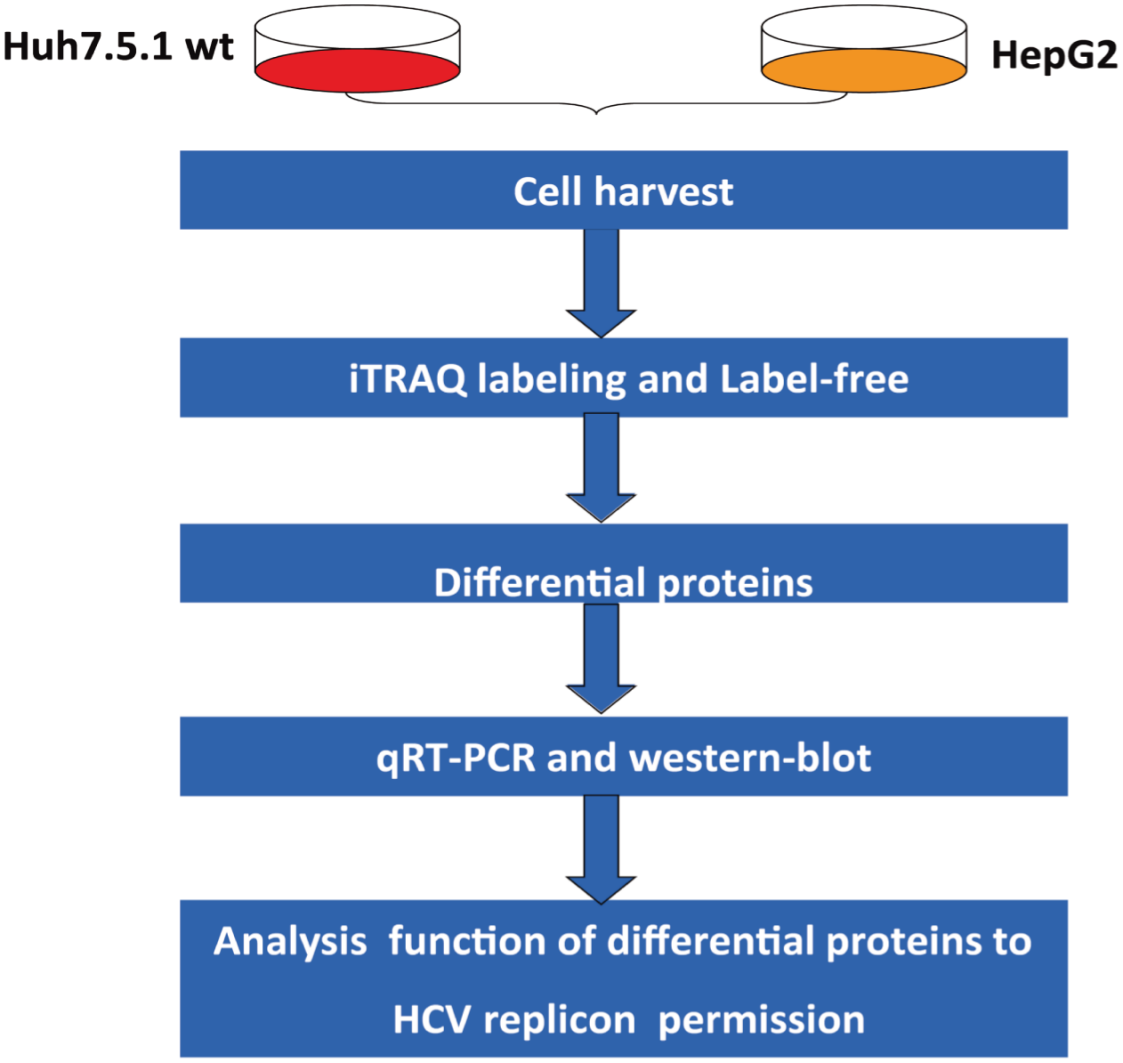
The overall workflow. Huh7.5.1 wt and HepG2 whole-cell proteins were harvested and analyzed using the Label-free and iTRAQ proteomic approaches, and the differential proteins were verified by qRT-PCR and western blot. The functions of selected differential proteins in HCV replication were analyzed further.

The inter-sample analysis is visualized using a volcano plot comparing the fold change and t-test p-value (Fig 1). A total of 393 proteins were determined to be modulated more than 2-fold with t-test p-value less than 0.05 (S1 Table).

#### iTRAQ-labeling approach

The mixed sample from HepG2 and Huh7.5.1 wt cells was added as an internal standard. After mixing with labeled HepG2 and Huh7.5.1 wt samples, the mixture was analyzed by the RP-RP LC/MS method. The RP-RPLC system operated at different pH values provides a higher practical peak capacity, and is commonly used for proteomic analysis [36–38]. In this study, iTRAQ labeled tryptic peptides were separated using first dimensional RPLC at high pH (pH = 10) into 60 fractions, which were concatenated into 20 fractions by combining fractions 1, 21, 41, 2, 22, 42 and so on. The 20 pooled fractions were finally submitted for RPLC-MS/MS analysis at low pH (pH = 2). The 20 raw files were merged into one file for Mascot database search, and the result was filtered using Scaffold with a 1% FDR at the protein level with decoy database and 2 unique peptides for each protein.

**Fig 2:**
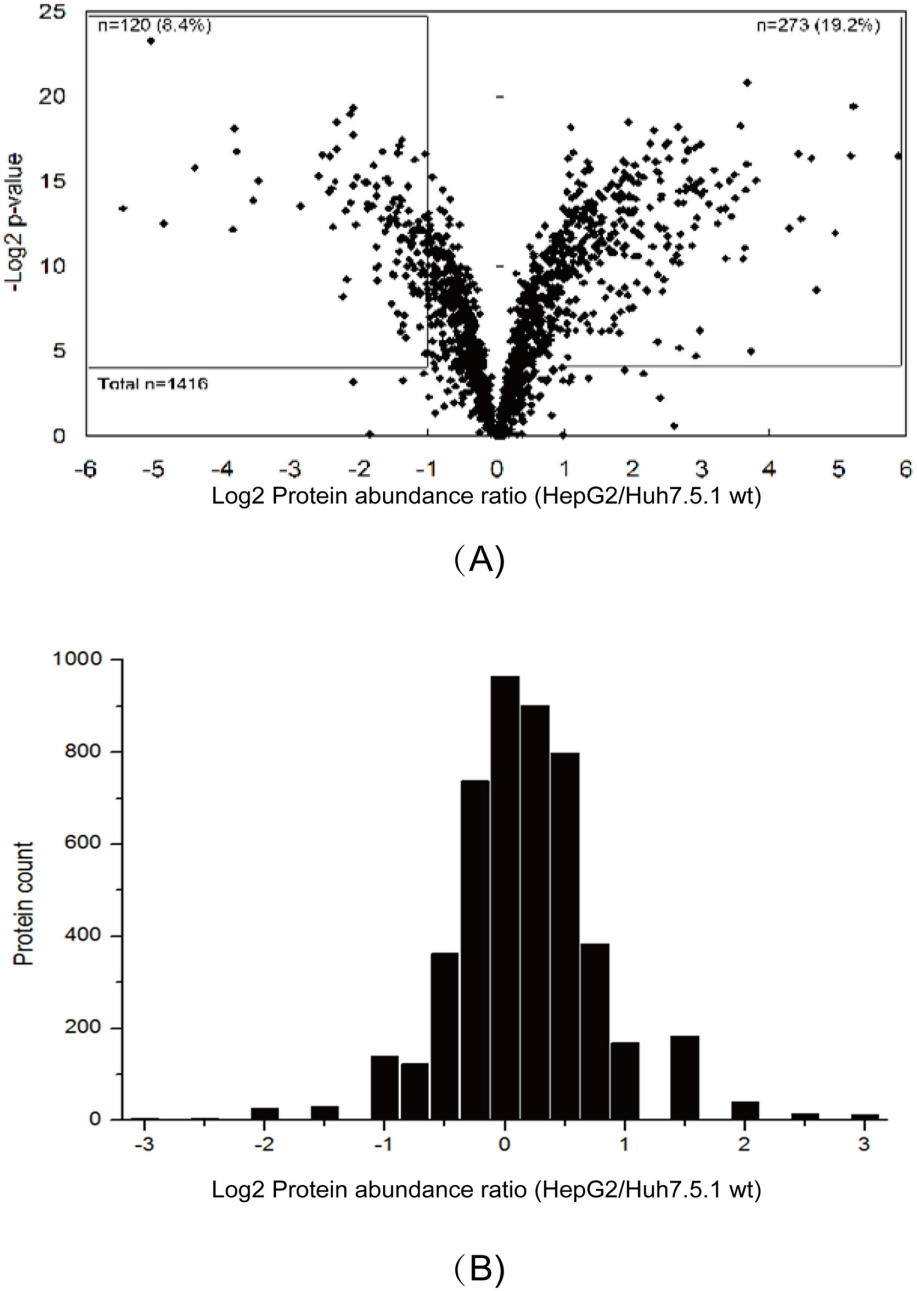
Quantitative analysis of proteins abundance changes detected using the label-free and iTRAQ approaches. (A) The volcano plot shows t-test p-values plotted against log2 values of protein fold changes. Data points in the lower center area of the plot have a log2 value close to 0 and a *p* value approaching 1 and indicate no significant change, whereas points in the upper left and upper right quadrants indicate significant negative and positive changes in protein abundance, respectively. (B) The iTRAQ ratio was transformed by log2 analysis. The proteins with abundance ratio within the range from -1 to +1 indicate no significant changes, and values larger than +1 or lower than -1 indicate significant positive and negative changes in protein abundance, respectively.

A total of 773,191 MS/MS were used for the database search and 215,287 spectra, 38,191 unique peptides and 4944 proteins were identified (Table 1, detailed data in S1 Table). From this dataset, 4900 proteins could be used for quantitative analysis. The protein abundance ratio distribution of the two cell lines is shown in Fig 2. The normal distribution of the protein abundance ratio and the greater than 90% ratio between -1 to +1 indicated that the iTRAQ-labeling approach provided good quantitative information. The differential proteins with over 10 fold change were manually validated and found they were artifacts by data processing, therefore, they were excluded from the final list. Total 451 differential proteins were found (S2 Table), 201 protein up-regulated and 250 down-regulated.

### Combination of label-free and iTRAQ-labeling approaches

When combined the results from the label-free and iTRAQ-labeling approaches were combined, a total of 730 differentially expressed proteins were identified. Among them 114 were regarded as differential by both approaches, and showed the same expression trend. A total of 56 proteins were identified as differential with one approach (44 and 12 from the label-free and iTRAQ approaches, respectively), but the other approach showed the reverse trend. To exclude false positive identification, these proteins were excluded from the following functional analyses. Therefore, a total of 674 proteins were considered as differentially expressed proteins for functional analysis.

### GO analysis

**Fig 3:**
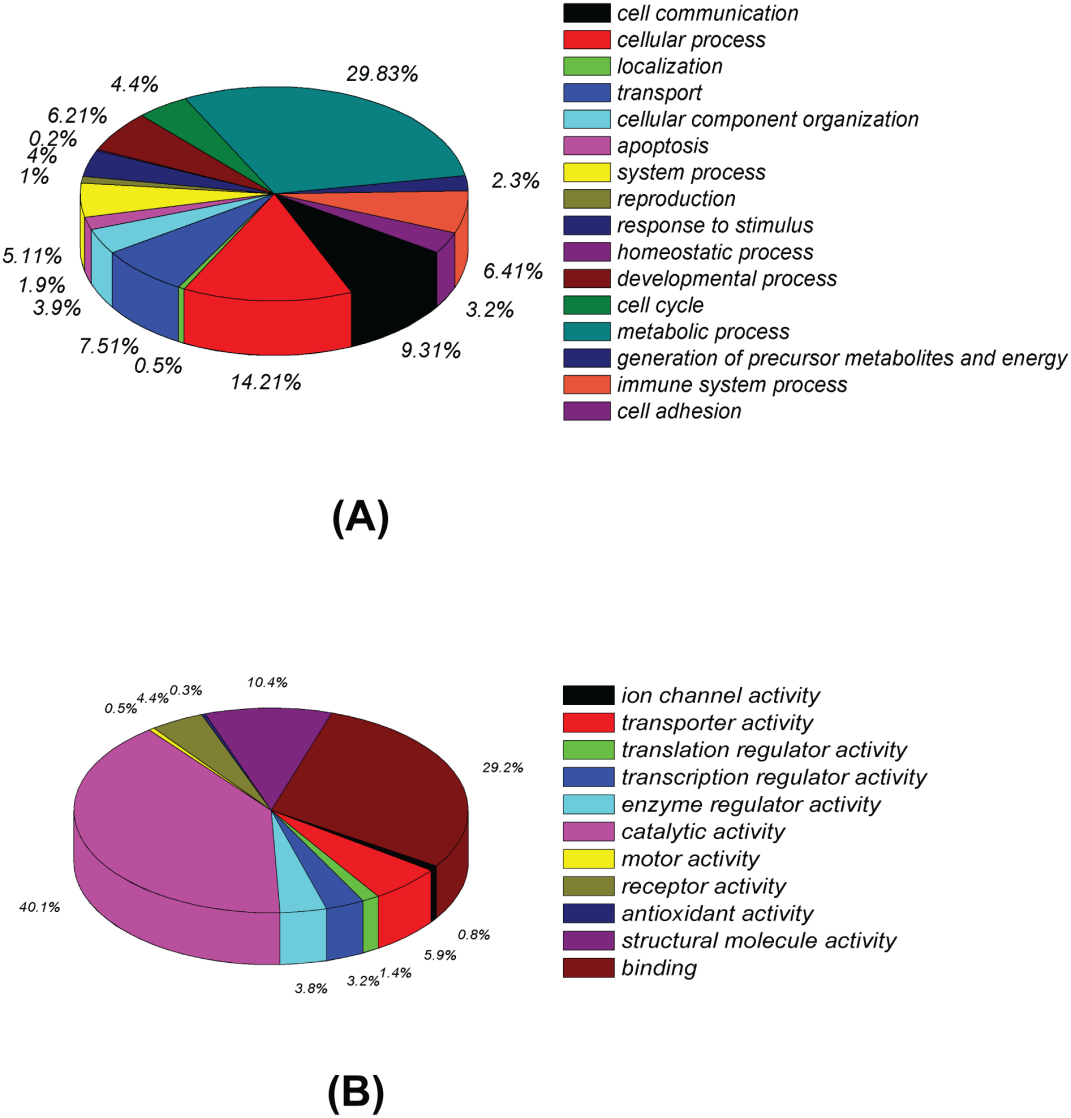
Classification of differentially expressed proteins based on GO annotation. (A) Categorization based upon biological process analysis of total differential protein expression. (B) Categorization based upon molecular function analysis of total differential protein expression. The distribution frequencies in regard to the specified categories within the given charts are indicated in % of the total number of protein entries.

To gain a functional understanding of the possible roles of the differentially expressed proteins, GO annotation was performed using “biological process” and “molecular function”. The 674 differential proteins were clustered according to their biological processes and molecular function (Fig 3). Proteins were involved in several biological processes that are relevant for HCV infection, especially growth and inflammation. A significant number of identified proteins (29.8%) were engaged in metabolic process, while the next top five biological process categories were cellular process (14.2%), cell communication (9.3%), transport (7.5%), immune system process (6.4%) and developmental process (6.2%) (Fig 3A). In the molecular function distribution category, the majority of the proteins were associated with three functions: catalytic activity (40.1%), binding (29.2%) and structural molecule activity (10.4%) (Fig 3B).

### IPA network analysis

**Fig 4:**
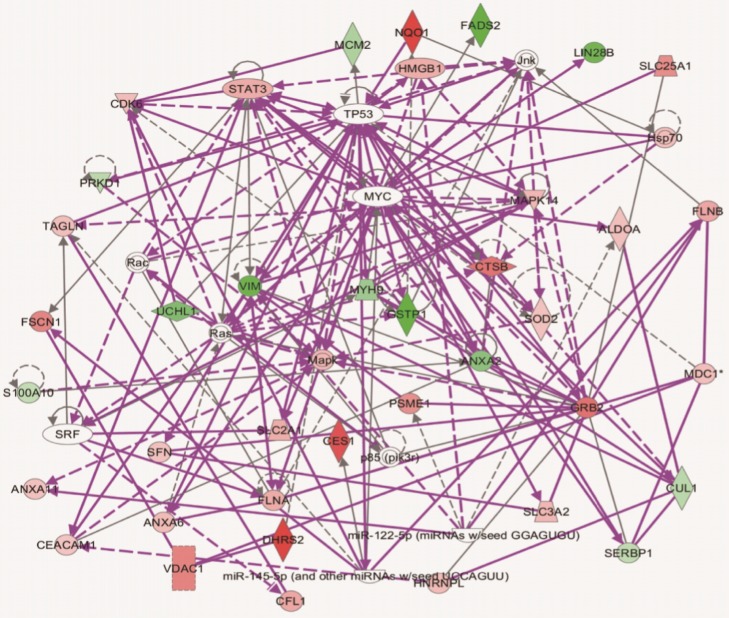
The interaction networks of differential proteins in Huh7.5.1 wt and HepG2 cell lines by IPA analysis. The networks of differentially expressed proteins related to Cancer, Gastrointestinal and Hepatic System Disease, Cell Death and Survival, Cellular Development, Growth and Proliferation. Proteins high in Huh7.5.1 wt cells and HepG2 cells are denoted in red and green, respectively. Any known direct connections between these proteins are indicated by solid lines, indirect interactions are shown with dashed lines.

Ingenuity Pathway Analysis (IPA) was used to determine whether proteins that changed in abundance could be mapped to specific functional networks. IPA analysis revealed that many of the differential proteins were located in networks of Cancer, Gastrointestinal and Hepatic System Disease (Fig 4). Several differential proteins including vimentin, CTSB, UCHL1, CES1, PSME1 and GSTP1, were key proteins in the merged network. These proteins interacted with each other or with important cell factors either directly or indirectly. Whether these networks target HCV or impact the permission of cells for HCV infection needed to elucidated.

### Validation of differential proteins by qRT-PCR and western blot analysis

**Fig 5:**
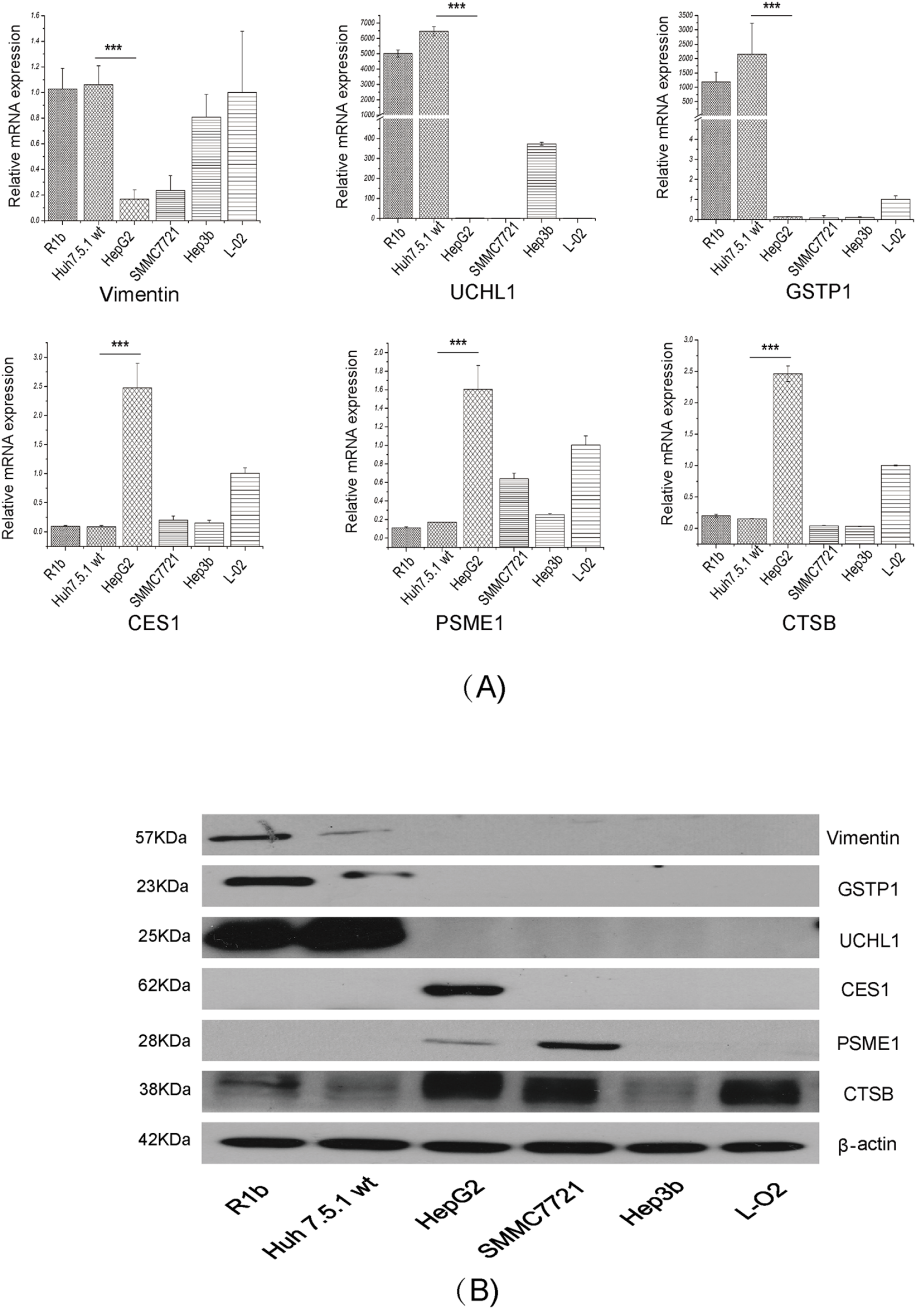
The key differentially expressed proteins in IPA networks analysis were validated by qRT-PCR and western blot. (A) The mRNA levels of six differential proteins in hepatic cell lines. (B) The protein levels of six key differential proteins in hepatic cell lines. NS3 was used as control for HCV-replicon expression for all the analyses, while GAPDH as control. And β-actin was used for protein loading control. *P<0.05, ** P<0.01 and ***P<0.001 versus negative control

A total of 37 differential proteins which were located in the merged networks or related with virus infection were selected for qRT-PCR analysis. The 37 proteins showed the same expression trend in qRT-PCR against proteomic analyses (S3 Fig). Among them, vimentin, PSME1, CES1, UCHL1, GSTP1 and CTSB were chosen for further study. Other hepatitis cell lines were used to better elucidate which proteins play a role in the HCV life cycle. However, the mRNA levels of GSTP1, UCHL1, and vimentin were extremely high in Huh7.5.1 wt cells compared to HepG2. While CES1, PSME1 and CTSB were identified with a high expression in HepG2 cells but not in R1b and Huh 7.5.1 wt cells (Fig 5A). All results consisted with the results both in label-free and iTRAQ.

To validate the protein level of above differential proteins, western blot was carried out to verify these results. Vimentin, GSTP1 and UCHL1 were highly expressed in R1b and Huh7.5.1 wt cells, while expression of PSME1, CES1 and CTSB were higher in HepG2 cells (Fig 5B). Expression of all 6 differentially expressed proteins in Huh7.5.1 wt and HepG2 is summarized in Table 3. Overall, the qRT-PCR and western blot analyses confirmed the results of the label-free and iTRAQ analysis.

**Table 3:** Identification of the 6 differential proteins in Huh7.5.1 wt and HepG2 cells detected by analysis of label-free, iTRAQ, qRT-PCR and western blot assays (Relative differential proteins: HepG2/Huh7.5.1 wt).

	label-free	iTRAQ	qRT-PCR	Western blot
GSTP1	0.0304	0.2000	0.0001	0.0604
UCHL1	0.0898	0.1053	0.0003	0.0205
vimentin	0.0700	0.1579	0.1588	0.2752
PSME1	5.3056	2.8000	9.3616	7.6806
CES1	21.5732	8.5000	28.4906	28.5858
CTSB	10.3232	2.8000	16.0261	2.2342

### Effects of GSTP1, UCHL1, vimentin, CES1, PMSE1 and CTSB on HCV replication

Although differentially expressed proteins were identified by both LC/MS/MS and western blot. The function of these proteins related to HCV infection still need to uncover. Vimentin, GSTP1 and UCHL1 exhibited high expression levels in Huh7.5.1 wt cells not in other hepatic cell lines, indicating that they might facilitate the permissiveness of the HCV replicon in differential cell lines.

**Fig 6:**
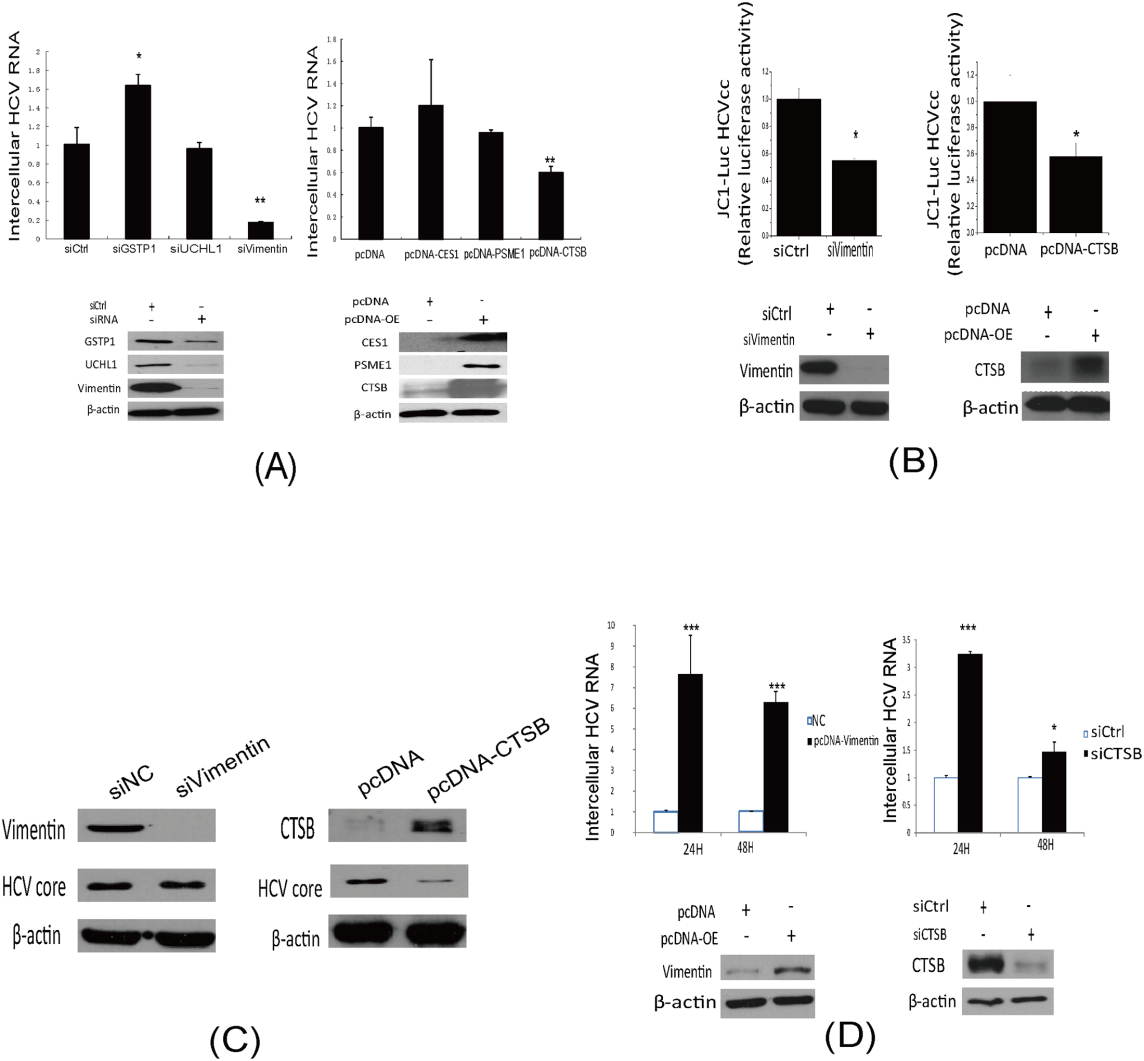
The effects of six key differential proteins on HCV RNA levels. (A) qRT-PCR analysis of HCV RNA levels by transfection of siRNA or over-expression of plasmids. (B) Luciferase assay of Jc1-Luc HCVcc in Huh7.5.1 cells transfected with siRNA or over-expression plasmids. (C) QRT-PCR analysis of HCV RNA levels in knockdown of CTSB or over-expression of vimentin in HepG2 cells after transfection with HCV replicon RNA. Western blot results showed the protein level of knockdown and over-expression differential proteins, and β-actin as protein loading control. (D) Huh7.5.1 cells were transfected with siRNA or over-expression plasmids of CTSB, then 24 h later, cells were infected with HCVcc (multiplicity of infection 0.1). At 48 h post infection, immunoblotting was performed with an anti-HCV core antibody. Each bar represents the average of triplicate data points with the standard deviation represented as the error bar. *P<0.05, ** P<0.01 and ***P<0.001 versus negative control

Knockdown of GSTP1, vimentin and UCHL1 in R1b cells were used to explore the differential proteins functions in HCV subgenome permission. Results illustrated that the RNA level of HCV highly decreased in vimentin knockdown cells, increased in GSTP1 knockdown cells and did not changed in UCHL1 knockdown cells (Fig 6A, left panel). The results indicate that vimentin might be associated with HCV replicon permissiveness.

And CES1, PSME1 and CTSB were over-expressed in R1b cells prior to detection HCV RNA. HCV replication did not change significantly in cells with over-expression of CES1 and PSME1. However, the HCV RNA level sharply decreased in cells with CTSB over-expression (Fig 6A, right panel), which indicated that CTSB might participate in HCV subgeonme permission in R1b cells. Effects of HCV to these proteins expression were illustrated, Vimentin, UCHL1, GSTP1 and CTSB were demonstrated decreased in HCV infection (S3 Fig).

Huh7.5.1 cells were infected with Jc1-Luc HCVcc after vimentin was knocked down or CTSB was over-expressed to further investigate roles of proteins on HCV RNA levels. Knockdown of vimentin or over-expression of CTSB both reduced luciferase activities (Fig 6B). The effect of CTSB and vimentin to viral protein expression were confirmed by HCV JFH1 infection. Results showed that CTSB inhibit viral protein expression, and vimentin didn’t show such effect (Fig 6C). And CTSB and vimentin didn’t affect R1b cell viability and growth in this study (data not show).

To verify the function of vimentin and CTSB to HCV RNA replication in HepG2 cell lines. Knockdown of CTSB or over-expression of vimentin was performed in HepG2 cells after the HCV subgenome RNA transfection. HCV RNA levels were analyzed at 24 h and 48 h after transfection. The results showed that levels of HCV RNA in treated cells were significant depleted slowly compared with the negative control (Fig 6D). Western blot analysis demonstrated knockdown or over-expression of indicated proteins level compared with the negative control (Fig 6A, 6B and 6C, down panel). These results may suggest that CTSB and vimentin participated in HCV RNA replication procession.

## Discussion

Recent advances in both proteomic methodologies [39–41] and cell culture models of HCV infection [3] make it possible to perform global characterization of the host cell protein response within the context of the complete set of HCV genes *in vitro*. Global characterization of the host cellular response to HCV has been developed using liquid chromatographic (LC) separations coupled with mass spectrometry (MS) for proteome analysis to identify potential gene markers of HCV-associated liver disease [42]. These reports were mainly based on the HCV replicon cell culture systems and have been particularly limited for several reasons. One limitation is that HCV replicon sequences contain many sequences absent from the HCV genome that are required to sustain the replication of the replicon. Additionally, the G418 selection required to select cells containing the HCV replicon influences the protein profile of host cells and results changes in the host cell proteome. These defects may result in identification of false positive proteins during proteome analysis that are independent of the HCV life cycle. We compared proteomic differences between two naïve hepatoma cell lines that showed significant differences in permissiveness for the HCV replicon. The use of naïve cells allowed us to avoid the influence of extrinsic factors and was favorable for selecting real HCV-related proteins.

Proteomics is a powerful tool for biological and medical analysis and has been successfully utilized in various fields. In this paper, we employed two quantitative proteomic methods, label-free (peptide peak area) and labeled (iTRAQ). The label-free approach is based on peptide peak area and provides comprehensive proteomic profile and quantitative results. It required high reproducibility of sample processing and LC-MS/MS results [43]. With the increase in sample number and multiple-dimensional separation, the variance of fold change will increase. Therefore, in this study 1D LC-MS/MS was used for label-free quantitative analysis to obtain more accurate quantitative results. The iTRAQ-labeling approach used an isotope labeling method and provided reliable quantitative information. However, protein ratio accuracy was often underestimated due to contamination of peptides with similar m/z’s included in the isolation window during ion selection and prior to MS/MS fragmentation. A previous study reported that prefractionation of samples could increase the proteome coverage [44]. In this study RP-RP 2D LC-MS/MS was adopted for the iTRAQ-labeling approach to obtain more comprehensive quantitative results. The combination of these two approaches not only provided more comprehensive quantitative information about the sample, but also mutually confirmed the results. The label-free approach provides more accurate quantitative information that may be under-estimated by the iTRAQ-labeling approach, while the iTRAQ-labeling approach using 2D LC-MS/MS provided higher proteome coverage that might be absent in 1D LC-MS/MS label-free analysis. Finally a comprehensive differential protein map (674 proteins) was presented.

Entry factors, immune defense, host factors are crucial for virus life cycle. It’s well known that the SCARB1, CD81, Claudin-1, OCLN are necessary for HCV to enter host cells, which all fully discussed [45–49]. And host cellular factors also affect virus tropism, while apolipoprotein E (apoE) was reported could specifically blocked the entry of HCV [50]. And there are other cellular factors required for HCV replication, cdc42 and other cellular factors proved to participate in HCV replication by interacting with HCV NS5A protein [51]. Recently, there are functional genomics studies revealed the extensive network of host for HCV life cycle. By small interfering RNA screening, lots of cellular factors participate in complete HCV replication cycle [52]. MiR-122 was reported to have crucial role in HCV replication, by protecting the viral genome from degradation. When ectopic expression miR-122 in other cell lines (HepG2, HEK293, mouse embryonic fibroblasts), the HCV RNA level were all highly enhanced. There is one question that the HCV RNA level still low in HepG2 cells after ectopic expression of miR-122 when compared with Huh7 cell lines [20, 53, 54].

The aim of this study was to identify proteins involved cell permissive, some of proteins have been reported to be relevant to HCV or HCV associated hepatocellular carcinoma. Creatine kinase B (CKB) is a key ATP-generating enzyme, was reported to be important for efficient replication of the HCV genome and propagation of infectious virus [55]. And in our data, CKB is richly expressed in Huh7.5.1 wt cells which can partly explain why the permission of HCV subgeonme in HepG2 is low. UCHL1, GSTP1 and CTSB, were linked to the TP53 signaling cascades, which has been suggested to be involved in carcinogenic processes and transcription regulation. TP53 mutation has been demonstrated to frequently exist in hepatocellular carcinoma patients infected with HCV [56]. UCHL1 is selectively expressed in the testis/ovary and brain and plays an important role in targeting of abnormal proteins for degradation through the ubiquitin-proteasome system [57, 58]. UCHL1 is particularly high expressed in Huh7.5.1 wt related cell lines, but loss of function in R1b cells indicated that it did not have an effect on HCV replication. And GSTP1 is an enzyme that catalyzes the conjugation of glutathione (GSH) to a variety of electrophilic substances. It has been reported that the GSTP1 gene is hypermethylated in genotype 1b HCV core protein-positive cells [59] and in hepatocellular carcinoma (HCC) tumors associated with HCV infection [60]. We discovered that knockdown of the GSTP1 led to an increase in HCV RNA levels in R1b cells. The association GSTP1 hepatocellular carcinoma indicates that it may function in the HCV life cycle [61]. We speculate that GSTP1 may be a cellular factor that acts as an antiviral element during HCV replication.

Vimentin is a type III intermediate filament (IF) protein expressed in mesenchymal cells, that is often used as a marker of mesenchymal derived cells or cells undergoing an epithelial-to-mesenchymal transition (EMT), and was reported participated in virus infection. During Dengue virus infection, vimentin interacts with the NS4A protein, and facilitates formation of the replication complex, suggesting that it participates in virus replication [62]. Vimentin expression is increased in HCV-infected human hepatoma cells compared with parental cells but not in patients without chronic hepatitis [63, 64]. In this study, knockdown of vimentin in R1b cells significantly impaired the RNA level of HCV in these cells, suggesting an effect to HCV replication (Fig 6A left panel). Moreover, over-expression of vimentin in HepG2 cells facilitates the survival of transiently transfected HCV RNA in the cells, which indicated a potential role that contributes to permissiveness (Fig 6D). However, vimentin didn’t affect replication of HCV while in HCV infection (Fig 6C), the exactly mechanisms vimentin affects HCV RNA replication remains to be elucidated. It had been reported that the HCV core protein level was affected by cellular vimentin content, and vimentin might enable the control HCV of production [65]. As vimentin is an intermediate filament-protein, we hypothesis that the replication process of HCV might draw support from intermediate filaments to complete its physiological activity.

Proteins enriched in HepG2 cells may be potential candidates for suppression of HCV replication or inhibition of the permissiveness for the HCV subgenome replicon. Major vault protein (MPV), is a virus-induced host factor that can suppress HCV replication by up-regulating type-I interferon production and up-regulating cellular antiviral responses [66]. This is consistent with our results that detected high levels of protein expression in HepG2 cells with a low permissiveness for the HCV replicon. CES1 is a protein involved in processing of triglycerides and cholesterol, and has been proven to facilitate HCV propagation [67]. In this study, over-expression of CES1 did not significantly change the level of HCV replication. And PSME1 were reported to have interaction with miR-122 which has been reported to facilitate HCV replication [68]. In our study the HCV RNA level was unaffected by PSME1 over-expression (Fig 6A). Taken together, these results might suggest that CES1 and PSME1 do not directly affect HCV replication.

CTSB, a lysosomal cysteine protease, is a candidate for an apoptotic mediator originating from acidic vesicles, induces apoptosis by enhancing mitochondrial release of cytochrome c and subsequent caspase activation in TNF-α treated hepatocytes [69]. We discovered that in R1b cells, the expression level of CTSB was greatly inhibited. Over-expression of CTSB led to a significant decrease of HCV RNA level and Jc1 luciferase activity in R1b cells, while knockdown of CTSB in HepG2 cells contributed to HCV RNA accumulation in the cells following transient transfection (Fig 6). These results suggested a close relationship between CTSB and HCV replication. It was reported that HCV, including HCV non-structural proteins, takes advantage of many pathways to avoid cellular apoptosis for survival and persistent propagation [70, 71]. And these results may partly explain how CTSB affects HCV replication. However, whether HCV has a direct or indirect effect on CTSB need more investigation.

In summary, we presented here a large-scale proteomic analysis of the HCV replicon in high-permissive and low-permissive cell lines for *in vitro* analysis of HCV replication. The results represented a comprehensive protein database of human cell lines, providing a baseline for characterization of HCV-related proteins in the cellular environment and, identification of potential targets for HCV replication and antiviral treatment. These results further provide the understanding of the molecular mechanism of cellular events and pathogenesis associated with HCV progression, which is useful for the identification of HCV infection-associated proteins.

## Supporting Information

S1 Fig Comparison of protein abundance for replicate experiments of Huh7.5.1 wt (A-C) and HepG2 (D-F) cells using the label-free approach. (TIF)

S2 Fig The 31 mRNA levels in Huh7.5.1 wt cells and HepG2 cells. The upper graph shows proteins rich in Huh7.5.1 wt cells. The under graph shows proteins rich in HepG2 cells.
(TIF)

S3 Fig The HCV effect to 6 differential proteins expression in Huh7.5.1 cells. (TIF)

S1 Table The protein and peptide list from the label-free method and 4-plex iTRAQ method. (XLSX)

S2 Table The differentially expressed protein list from both the label-free and 4-plex iTRAQ quantitation methods. (XLSX)

S3 Table The results and primers of differential proteins expression by analysis of qRT- PCR. (XLSX)

## References

[1] Moradpour D, Penin F, Rice CM. Replication of hepatitis C virus Nature reviews Microbiology 2007 p. 453–63. 10.1038/nrmicro1645 17487147

[2] Bartenschlager R, Sparacio S. Hepatitis C virus molecular clones and their replication capacity in vivo and in cell culture. Virus research. 2007;127(2):195–207. 10.1016/j.virusres.2007.02.022 . 17428568

[3] Lohmann V, Korner F, Koch J, Herian U, Theilmann L, Bartenschlager R. Replication of subgenomic hepatitis C virus RNAs in a hepatoma cell line. Science. 1999;285(5424):110–3. . 10390360 10.1126/science.285.5424.110

[4] Bartenschlager R, Lohmann V. Replication of hepatitis C virus. The Journal of general virology. 2000;81(Pt 7):1631–48. . 10859368 10.1099/0022-1317-81-7-1631

[5] Blight KJ, McKeating JA, Marcotrigiano J, Rice CM. Efficient replication of hepatitis C virus genotype 1a RNAs in cell culture. Journal of virology. 2003;77(5):3181–90. 12584342 10.1128/JVI.77.5.3181-3190.2003 PMC149761

[6] Gu B, Gates AT, Isken O, Behrens SE, Sarisky RT. Replication studies using genotype 1a subgenomic hepatitis C virus replicons. Journal of virology. 2003;77(9):5352–9. 12692237 10.1128/JVI.77.9.5352-5359.2003 PMC153987

[7] Kato T, Date T, Miyamoto M, Furusaka A, Tokushige K, Mizokami M, et al Efficient replication of the genotype 2a hepatitis C virus subgenomic replicon. Gastroenterology. 2003;125(6):1808–17. . 14724833 10.1053/j.gastro.2003.09.023

[8] Kato T, Furusaka A, Miyamoto M, Date T, Yasui K, Hiramoto J, et al Sequence analysis of hepatitis C virus isolated from a fulminant hepatitis patient. Journal of medical virology. 2001;64(3):334–9. . 11424123 10.1002/jmv.1055

[9] Date T, Kato T, Miyamoto M, Zhao Z, Yasui K, Mizokami M, et al Genotype 2a hepatitis C virus subgenomic replicon can replicate in HepG2 and IMY-N9 cells. The Journal of biological chemistry. 2004;279(21):22371–6. 10.1074/jbc.M311120200 . 14990575

[10] Kato T, Date T, Miyamoto M, Zhao Z, Mizokami M, Wakita T. Nonhepatic cell lines HeLa and 293 support efficient replication of the hepatitis C virus genotype 2a subgenomic replicon. Journal of virology. 2005;79(1):592–6. 10.1128/JVI.79.1.592-596.2005 15596851 PMC538706

[11] Ali S, Pellerin C, Lamarre D, Kukolj G. Hepatitis C virus subgenomic replicons in the human embryonic kidney 293 cell line. Journal of virology. 2004;78(1):491–501. 14671129 10.1128/JVI.78.1.491-501.2004 PMC303421

[12] Wakita T, Pietschmann T, Kato T, Date T, Miyamoto M, Zhao Z, et al Production of infectious hepatitis C virus in tissue culture from a cloned viral genome. Nature medicine. 2005;11(7):791–6. 10.1038/nm1268 15951748 PMC2918402

[13] Zhong J, Gastaminza P, Cheng G, Kapadia S, Kato T, Burton DR, et al Robust hepatitis C virus infection in vitro. Proceedings of the National Academy of Sciences of the United States of America. 2005;102(26):9294–9. 10.1073/pnas.0503596102 15939869 PMC1166622

[14] Gottwein JM, Bukh J. Cutting the gordian knot-development and biological relevance of hepatitis C virus cell culture systems. Advances in virus research. 2008;71:51–133. 10.1016/S0065-3527(08)00002-X . 18585527

[15] Kato N, Mori K, Abe K, Dansako H, Kuroki M, Ariumi Y, et al Efficient replication systems for hepatitis C virus using a new human hepatoma cell line. Virus research. 2009;146(1–2):41–50. 10.1016/j.virusres.2009.08.006 . 19720094

[16] Kambara H, Fukuhara T, Shiokawa M, Ono C, Ohara Y, Kamitani W, et al Establishment of a novel permissive cell line for the propagation of hepatitis C virus by expression of microRNA miR122. Journal of virology. 2012;86(3):1382–93. 10.1128/JVI.06242-11 22114337 PMC3264374

[17] Blight KJ, McKeating JA, Rice CM. Highly permissive cell lines for subgenomic and genomic hepatitis C virus RNA replication. Journal of virology. 2002;76(24):13001–14. 12438626 10.1128/JVI.76.24.13001-13014.2002 PMC136668

[18] Lohmann V, Hoffmann S, Herian U, Penin F, Bartenschlager R. Viral and cellular determinants of hepatitis C virus RNA replication in cell culture. Journal of virology. 2003;77(5):3007–19. 12584326 10.1128/JVI.77.5.3007-3019.2003 PMC149776

[19] Jopling CL, Yi M, Lancaster AM, Lemon SM, Sarnow P. Modulation of hepatitis C virus RNA abundance by a liver-specific MicroRNA. Science. 2005;309(5740):1577–81. 10.1126/science.1113329 . 16141076

[20] Narbus CM, Israelow B, Sourisseau M, Michta ML, Hopcraft SE, Zeiner GM, et al HepG2 cells expressing microRNA miR-122 support the entire hepatitis C virus life cycle. Journal of virology. 2011;85(22):12087–92. 10.1128/JVI.05843-11 21917968 PMC3209320

[21] Jacobs JM, Diamond DL, Chan EY, Gritsenko MA, Qian W, Stastna M, et al Proteome analysis of liver cells expressing a full-length hepatitis C virus (HCV) replicon and biopsy specimens of posttransplantation liver from HCV-infected patients. Journal of virology. 2005;79(12):7558–69. 10.1128/JVI.79.12.7558-7569.2005 15919910 PMC1143647

[22] XUN Meng Z S-h, CAO Chun-xia, SONG Juan, SHAO Ming-ming, CHU Yong-lie. Proteomic analysis of HuH-7 cells harboring in vitro-transcribed fulllength hepatitis C virus 1b RNA. Acta pharmacologica Sinica. 2008;29(6):720–7. 10.1111/j.1745-7254.2008.00789.x 18501119

[23] Harris D, Zhang Z, Chaubey B, Pandey VN. Identification of cellular factors associated with the 3'-nontranslated region of the hepatitis C virus genome. Molecular & cellular proteomics: MCP. 2006;5(6):1006–18. 10.1074/mcp.M500429-MCP200 . 16500930

[24] Tingting P, Caiyun F, Zhigang Y, Pengyuan Y, Zhenghong Y. Subproteomic analysis of the cellular proteins associated with the 3' untranslated region of the hepatitis C virus genome in human liver cells. Biochemical and biophysical research communications. 2006;347(3):683–91. 10.1016/j.bbrc.2006.06.144 . 16842740

[25] Miao R, Wei J, Zhang Q, Sajja V, Yang J, Wang Q. Redifferentiation of human hepatoma cells (SMMC-7721) induced by two new highly oxygenated bisabolane-type sesquiterpenes. Journal of biosciences. 2008;33(5):723–30. . 19179760 10.1007/s12038-008-0092-x

[26] Wu JM L J, Zhang L, Liang KH. Establishment and identification of LO2 cell line transfected by HBV genome. Zhonghua Gan Zang Bing Za Zhi. 2005;13(9):702–3. 16174468

[27] Xin Z, Han W, Zhao Z, Xia Q, Yin B, Yuan J, et al PCBP2 enhances the antiviral activity of IFN-alpha against HCV by stabilizing the mRNA of STAT1 and STAT2. PloS one. 2011;6(10):e25419 10.1371/journal.pone.0025419 22022391 PMC3191149

[28] Wisniewski JR, Zougman A, Nagaraj N, Mann M. Universal sample preparation method for proteome analysis. Nature methods. 2009;6(5):359–62. 10.1038/nmeth.1322 . 19377485

[29] Ishii T, Teramoto S, Matsuse T. GSTP1 affects chemoresistance against camptothecin in human lung adenocarcinoma cells. Cancer letters. 2004;216(1):89–102. 10.1016/j.canlet.2004.05.018 . 15500952

[30] Bheda A, Shackelford J, Pagano JS. Expression and functional studies of ubiquitin C-terminal hydrolase L1 regulated genes. PloS one. 2009;4(8):e6764 10.1371/journal.pone.0006764 19707515 PMC2729380

[31] Mak TN, Fischer N, Laube B, Brinkmann V, Metruccio MM, Sfanos KS, et al Propionibacterium acnes host cell tropism contributes to vimentin-mediated invasion and induction of inflammation. Cellular microbiology. 2012;14(11):1720–33. 10.1111/j.1462-5822.2012.01833.x . 22759266

[32] Cheng M, Si Y, Niu Y, Liu X, Li X, Zhao J, et al High-throughput profiling of alpha interferon- and interleukin-28B-regulated microRNAs and identification of let-7s with anti-hepatitis C virus activity by targeting IGF2BP1. Journal of virology. 2013;87(17):9707–18. 10.1128/JVI.00802-13 23824794 PMC3754137

[33] Liu X, Huang Y, Cheng M, Pan L, Si Y, Li G, et al Screening and rational design of hepatitis C virus entry inhibitory peptides derived from GB virus A NS5A. Journal of virology. 2013;87(3):1649–57. 10.1128/JVI.02201-12 23175359 PMC3554153

[34] Morchang A, Panaampon J, Suttitheptumrong A, Yasamut U, Noisakran S, Yenchitsomanus PT, et al Role of cathepsin B in dengue virus-mediated apoptosis. Biochemical and biophysical research communications. 2013;438(1):20–5. 10.1016/j.bbrc.2013.07.009 . 23867824

[35] Waggoner SA, Johannes GJ, Liebhaber SA. Depletion of the poly(C)-binding proteins alphaCP1 and alphaCP2 from K562 cells leads to p53-independent induction of cyclin-dependent kinase inhibitor (CDKN1A) and G1 arrest. The Journal of biological chemistry. 2009;284(14):9039–49. 10.1074/jbc.M806986200 19211566 PMC2666552

[36] Gilar M, Olivova P, Daly AE, Gebler JC. Orthogonality of separation in two-dimensional liquid chromatography. Analytical chemistry. 2005;77(19):6426–34. 10.1021/ac050923i . 16194109

[37] Wang Y, Yang F, Gritsenko MA, Wang Y, Clauss T, Liu T, et al Reversed-phase chromatography with multiple fraction concatenation strategy for proteome profiling of human MCF10A cells. Proteomics. 2011;11(10):2019–26. 10.1002/pmic.201000722 21500348 PMC3120047

[38] Song C, Ye M, Han G, Jiang X, Wang F, Yu Z, et al Reversed-phase-reversed-phase liquid chromatography approach with high orthogonality for multidimensional separation of phosphopeptides. Analytical chemistry. 2010;82(1):53–6. 10.1021/ac9023044 . 19950968

[39] Casado-Vela J, Martinez-Esteso MJ, Rodriguez E, Borras E, Elortza F, Bru-Martinez R. iTRAQ-based quantitative analysis of protein mixtures with large fold change and dynamic range. Proteomics. 2010;10(2):343–7. 10.1002/pmic.200900509 . 20029838

[40] Bouchal P, Roumeliotis T, Hrstka R, Nenutil R, Vojtesek B, Garbis SD. Biomarker discovery in low-grade breast cancer using isobaric stable isotope tags and two-dimensional liquid chromatography-tandem mass spectrometry (iTRAQ-2DLC-MS/MS) based quantitative proteomic analysis. Journal of proteome research. 2009;8(1):362–73. 10.1021/pr800622b . 19053527

[41] Zhu M, Dai S, McClung S, Yan X, Chen S. Functional differentiation of Brassica napus guard cells and mesophyll cells revealed by comparative proteomics. Molecular & cellular proteomics: MCP. 2009;8(4):752–66. 10.1074/mcp.M800343-MCP200 19106087 PMC2667361

[42] Kim W, Lim SO, Kim JS, Ryu YH, Byeon JY, Kim HJ, et al Comparison of proteome between hepatitis B virus- and hepatitis C virus-associated hepatocellular carcinoma. Clinical Cancer Research. 2003;9(15):5493–500. WOS:000187014200009. 14654528

[43] Zhu W, Smith JW, Huang CM. Mass spectrometry-based label-free quantitative proteomics. Journal of biomedicine & biotechnology. 2010;2010:840518 10.1155/2010/840518 19911078 PMC2775274

[44] Wang HX, Alvarez S, Hicks LM. Comprehensive Comparison of iTRAQ and Label-free LC-Based Quantitative Proteomics Approaches Using Two Chlamydomonas reinhardtii Strains of Interest for Biofuels Engineering. Journal of proteome research. 2012;11(1):487–501. 10.1021/Pr2008225 WOS:000298827700042. 22059437

[45] Pileri P U Y, Campagnoli S, Galli G, Falugi F, Petracca R, Weiner AJ, Houghton M, Rosa D, Grandi G, Abrignani S. Binding of hepatitis C virus to CD81. science. 1998;282(5390):938–41. 10.1126/science.282.5390.938 9794763

[46] Scarselli E, Ansuini H, Cerino R, Roccasecca RM, Acali S, Filocamo G, et al The human scavenger receptor class B type I is a novel candidate receptor for the hepatitis C virus. Embo Journal. 2002;21(19):5017–25. 10.1093/Emboj/Cdf529 WOS:000178502600002. 12356718 PMC129051

[47] Aly HH, Watashi K, Hijikata M, Kaneko H, Takada Y, Egawa H, et al Serum-derived hepatitis C virus infectivity in interferon regulatory factor-7-suppressed human primary hepatocytes. Journal of hepatology. 2007;46(1):26–36. 10.1016/j.jhep.2006.08.018 . 17112629

[48] Evans MJ, von Hahn T, Tscherne DM, Syder AJ, Panis M, Wolk B, et al Claudin-1 is a hepatitis C virus co-receptor required for a late step in entry. Nature. 2007;446(7137):801–5. 10.1038/nature05654 . 17325668

[49] Ploss A, Evans MJ, Gaysinskaya VA, Panis M, You H, de Jong YP, et al Human occludin is a hepatitis C virus entry factor required for infection of mouse cells. Nature. 2009;457(7231):882–6. 10.1038/nature07684 19182773 PMC2762424

[50] Liu S, McCormick KD, Zhao W, Zhao T, Fan D, Wang T. Human apolipoprotein E peptides inhibit hepatitis C virus entry by blocking virus binding. Hepatology. 2012;56(2):484–91. 10.1002/hep.25665 22334503 PMC3362681

[51] Maqbool MA, Imache MR, Higgs MR, Carmouse S, Pawlotsky JM, Lerat H. Regulation of hepatitis C virus replication by nuclear translocation of nonstructural 5A protein and transcriptional activation of host genes. Journal of virology. 2013;87(10):5523–39. 10.1128/JVI.00585-12 23468497 PMC3648193

[52] Li Q, Zhang YY, Chiu S, Hu Z, Lan KH, Cha H, et al Integrative functional genomics of hepatitis C virus infection identifies host dependencies in complete viral replication cycle. PLoS pathogens. 2014;10(5):e1004163 10.1371/journal.ppat.1004163 24852294 PMC4095987

[53] Lin LT, Noyce RS, Pham TN, Wilson JA, Sisson GR, Michalak TI, et al Replication of subgenomic hepatitis C virus replicons in mouse fibroblasts is facilitated by deletion of interferon regulatory factor 3 and expression of liver-specific microRNA 122. Journal of virology. 2010;84(18):9170–80. 10.1128/JVI.00559-10 20592082 PMC2937658

[54] Chang J, Guo JT, Jiang D, Guo H, Taylor JM, Block TM. Liver-specific microRNA miR-122 enhances the replication of hepatitis C virus in nonhepatic cells. Journal of virology. 2008;82(16):8215–23. 10.1128/JVI.02575-07 18550664 PMC2519557

[55] Wyss M, Kaddurah-Daouk R. Creatine and creatinine metabolism. Physiological reviews. 2000;80(3):1107–213. . 10893433 10.1152/physrev.2000.80.3.1107

[56] Hussain SP, Schwank J, Staib F, Wang XW, Harris CC. TP53 mutations and hepatocellular carcinoma: insights into the etiology and pathogenesis of liver cancer. Oncogene. 2007;26(15):2166–76. 10.1038/sj.onc.1210279 . 17401425

[57] Yin L, Krantz B, Russell NS, Deshpande S, Wilkinson KD. Nonhydrolyzable diubiquitin analogues are inhibitors of ubiquitin conjugation and deconjugation. Biochemistry. 2000;39(32):10001–10. . 10933821 10.1021/bi0007019

[58] Kwon J, Wang YL, Setsuie R, Sekiguchi S, Sato Y, Sakurai M, et al Two closely related ubiquitin C-terminal hydrolase isozymes function as reciprocal modulators of germ cell apoptosis in cryptorchid testis. The American journal of pathology. 2004;165(4):1367–74. 10.1016/S0002-9440(10)63394-9 15466400 PMC1618639

[59] Ripoli M, Barbano R, Balsamo T, Piccoli C, Brunetti V, Coco M, et al Hypermethylated levels of E-cadherin promoter in Huh-7 cells expressing the HCV core protein. Virus research. 2011;160(1–2):74–81. 10.1016/j.virusres.2011.05.014 . 21640770

[60] Lambert MP, Paliwal A, Vaissiere T, Chemin I, Zoulim F, Tommasino M, et al Aberrant DNA methylation distinguishes hepatocellular carcinoma associated with HBV and HCV infection and alcohol intake. Journal of hepatology. 2011;54(4):705–15. 10.1016/j.jhep.2010.07.027 . 21146512

[61] Zhao Y, Wang Q, Deng X, Shi P, Wang Z. Quantitative assessment of the association between GSTP1 gene Ile105Val polymorphism and susceptibility to hepatocellular carcinoma. Tumour biology: the journal of the International Society for Oncodevelopmental Biology and Medicine. 2013;34(4):2121–6. 10.1007/s13277-013-0695-1 . 23765758

[62] Teo CS, Chu JJ. Cellular Vimentin Regulates Construction of Dengue Virus Replication Complexes through Interaction with NS4A Protein. Journal of virology. 2014;88(4):1897–913. 10.1128/JVI.01249-13 24284321 PMC3911532

[63] Ghosh S, Ahrens WA, Phatak SU, Hwang S, Schrum LW, Bonkovsky HL. Association of filamin A and vimentin with hepatitis C virus proteins in infected human hepatocytes. Journal of viral hepatitis. 2011;18(10):e568–77. 10.1111/j.1365-2893.2011.01487.x . 21914078

[64] Lee CF, Ling ZQ, Zhao T, Lee KR. Distinct expression patterns in hepatitis B virus- and hepatitis C virus-infected hepatocellular carcinoma. World journal of gastroenterology: WJG. 2008;14(39):6072–7. 18932288 10.3748/wjg.14.6072 PMC2760187

[65] Nitahara-Kasahara Y, Fukasawa M, Shinkai-Ouchi F, Sato S, Suzuki T, Murakami K, et al Cellular vimentin content regulates the protein level of hepatitis C virus core protein and the hepatitis C virus production in cultured cells. Virology. 2009;383(2):319–27. 10.1016/j.virol.2008.10.009 . 19013628

[66] Liu S, Hao Q, Peng N, Yue X, Wang Y, Chen Y, et al Major vault protein: a virus-induced host factor against viral replication through the induction of type-I interferon. Hepatology. 2012;56(1):57–66. 10.1002/hep.25642 . 22318991

[67] Blais DR, Lyn RK, Joyce MA, Rouleau Y, Steenbergen R, Barsby N, et al Activity-based Protein Profiling Identifies a Host Enzyme, Carboxylesterase 1, Which Is Differentially Active during Hepatitis C Virus Replication. Journal of Biological Chemistry. 2010;285(33):25602–12. 10.1074/jbc.M110.135483 WOS:000280682400054. 20530478 PMC2919124

[68] Diao S, Zhang JF, Wang H, He ML, Lin MC, Chen Y, et al Proteomic identification of microRNA-122a target proteins in hepatocellular carcinoma. Proteomics. 2010;10(20):3723–31. 10.1002/pmic.201000050 . 20859956

[69] Guicciardi ME, Deussing J, Miyoshi H, Bronk SF, Svingen PA, Peters C, et al Cathepsin B contributes to TNF-alpha-mediated hepatocyte apoptosis by promoting mitochondrial release of cytochrome c. The Journal of clinical investigation. 2000;106(9):1127–37. 10.1172/JCI9914 11067865 PMC301415

[70] Lan KH, Sheu ML, Hwang SJ, Yen SH, Chen SY, Wu JC, et al HCV NS5A interacts with p53 and inhibits p53-mediated apoptosis. Oncogene. 2002;21(31):4801–11. 10.1038/sj.onc.1205589 . 12101418

[71] Majumder M, Ghosh AK, Steele R, Zhou XY, Phillips NJ, Ray R, et al Hepatitis C virus NS5A protein impairs TNF-mediated hepatic apoptosis, but not by an anti-FAS antibody, in transgenic mice. Virology. 2002;294(1):94–105. 10.1006/viro.2001.1309 . 11886269

