# Supplementary figures and images for: Quantitative Proteomics Analysis of the Hepatitis C Virus Replicon High-Permissive and Low-Permissive Cell Lines
Source: PLoS One. 2015 Nov 6;10(11):e0142082. doi: 10.1371/journal.pone.0142082 (PMC4636247; doi:10.1371/journal.pone.0142082)

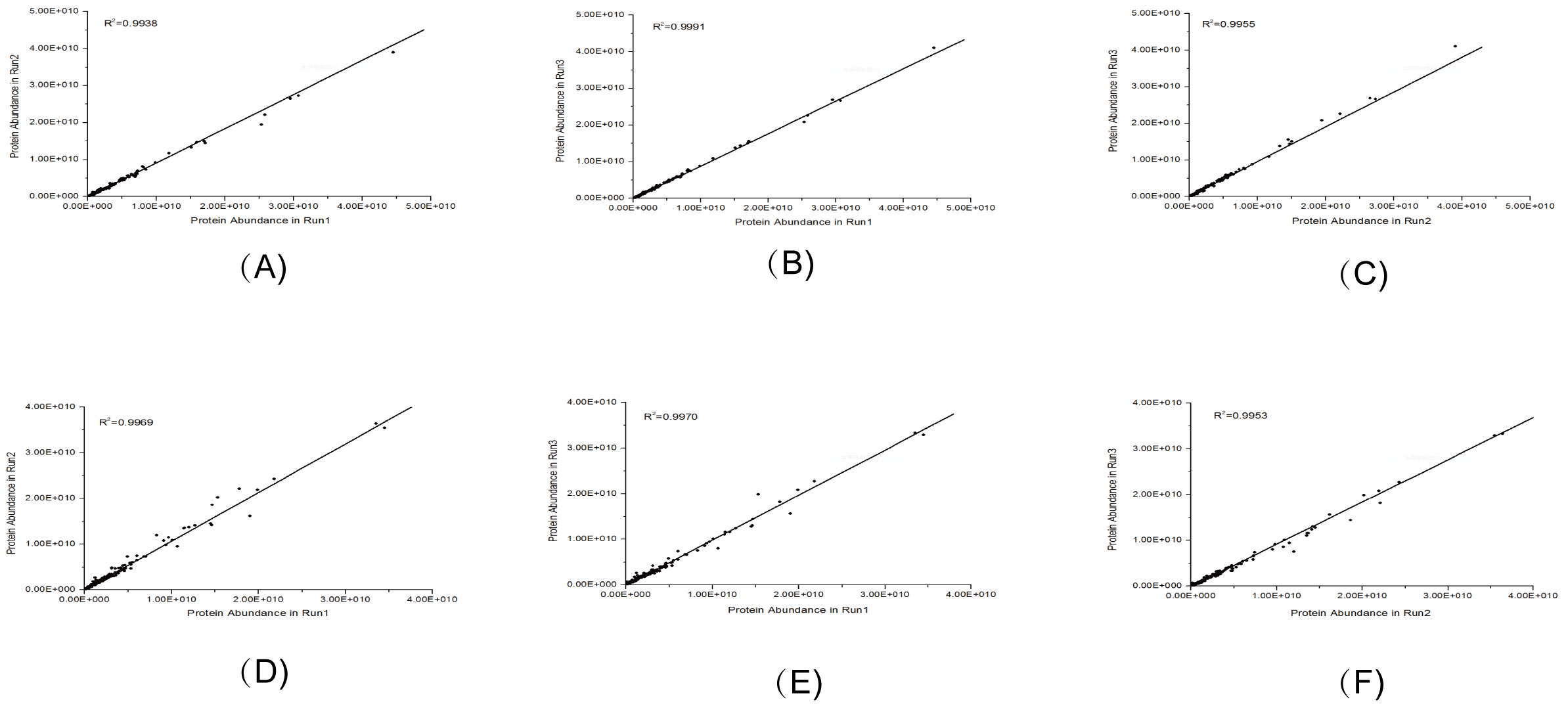

Supplement: S1 Fig — (TIF) [file pone.0142082.s001.tif]

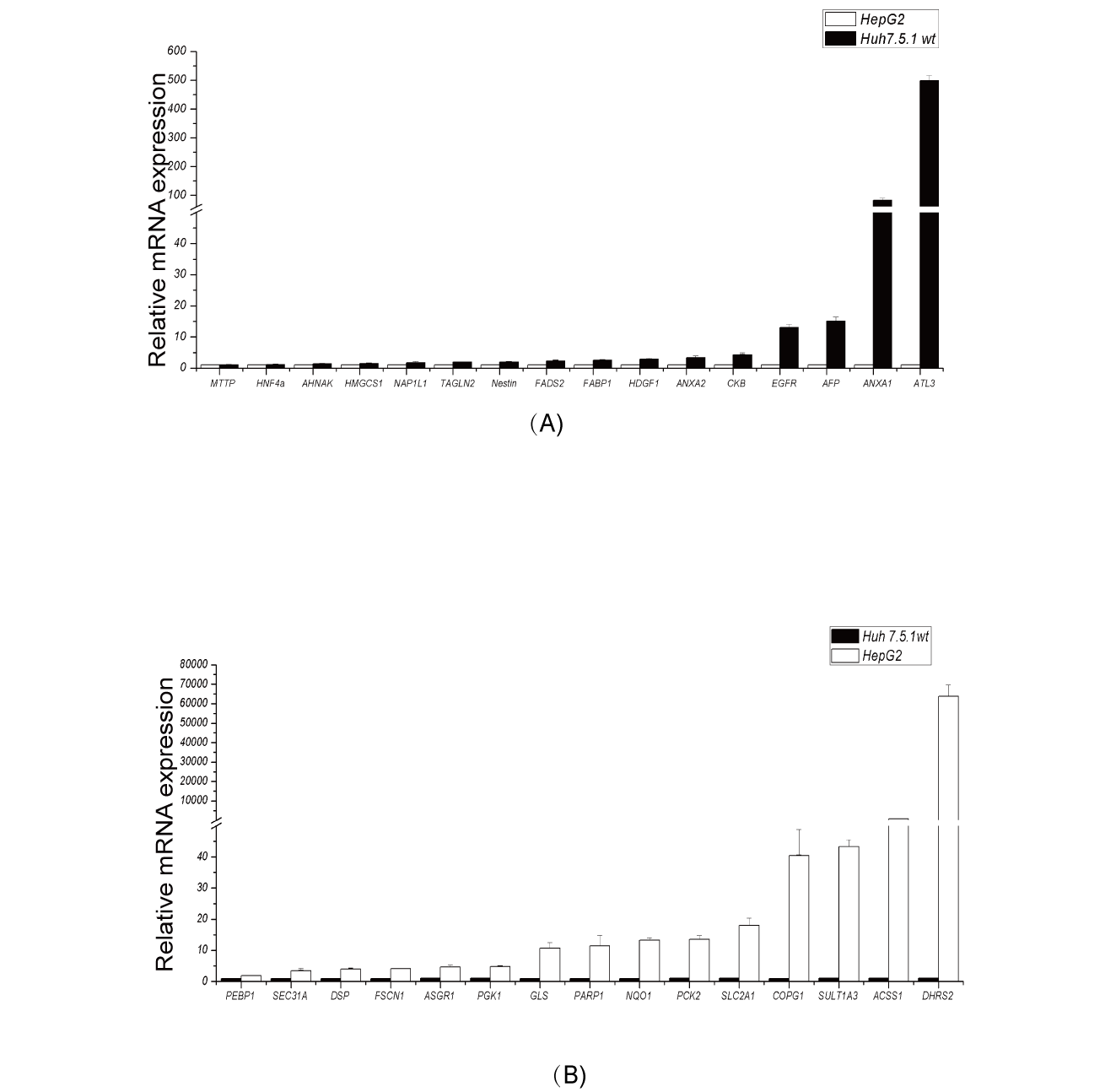

Supplement: S2 Fig — The upper graph shows proteins rich in Huh7.5.1 wt cells. The under graph shows proteins rich in HepG2 cells. (TIF) [file pone.0142082.s002.tif]

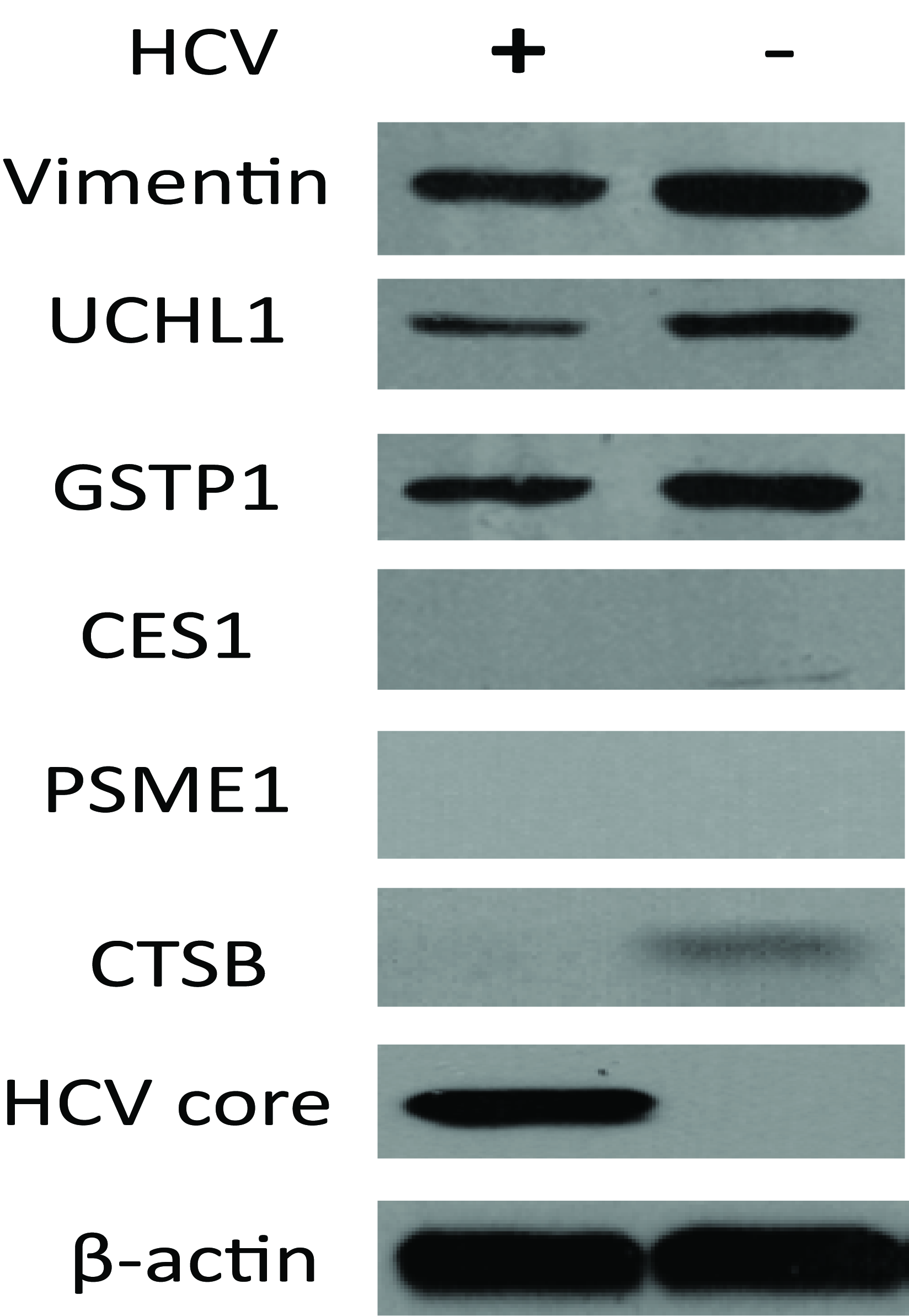

Supplement: S3 Fig — (TIF) [file pone.0142082.s003.tif]
